# Terahertz coherent receiver using a single resonant tunnelling diode

**DOI:** 10.1038/s41598-019-54627-8

**Published:** 2019-12-02

**Authors:** Yousuke Nishida, Naoki Nishigami, Sebastian Diebold, Jaeyoung Kim, Masayuki Fujita, Tadao Nagatsuma

**Affiliations:** 1 Graduate School of Engineering Science, Osaka University, 1-3 Machikaneyama, Toyonaka, Osaka 560-8531 Japan; 2 ROHM Co., Ltd., 21 Saiin Mizosaki, Ukyo, Kyoto 615-8585 Japan

**Keywords:** Electrical and electronic engineering, Electronic and spintronic devices

## Abstract

Towards exploring advanced applications of terahertz (THz) electromagnetic waves, great efforts are being applied to develop a compact and sensitive THz receiver. Here, we propose a simple coherent detection system using a single resonant tunnelling diode (RTD) oscillator through self-oscillating mixing with an RTD oscillator injection-locked by a carrier wave. Coherent detection is successfully demonstrated with an enhancement in the sensitivity of >20 dB compared to that of direct detection. As a proof of concept, we performed THz wireless communications using an RTD coherent receiver and transmitter. We achieved 30-Gbit/s real-time error-free transmission, which is the highest among all electronic systems without error correction to date. Our results show that the proposed system can reduce the size and power consumption of various THz systems including sensing, imaging and ranging, which would enable progress to be made in a wide range of fields in such as material science, medicine, chemistry, biology, physics, astronomy, security, robotics and motor vehicle.

## Introduction

Terahertz (THz) electromagnetic waves, with frequencies ranging from around 0.1 THz to 10 THz, have gained attention as the frontiers of electronics and photonics and now the subject of an interdisciplinary area of research. Unique potential applications of THz waves, such as high-resolution sensing and broadband communications, have been developed^1–8^. Electronic devices are prime candidates to make compact and low-power-consumption systems towards expanding such THz technologies to various fields. Recently, THz diodes such as impact ionisation avalanche transit-time diodes^9^, tunnelling transit-time diodes^10^, Gunn diodes^11^, resonant tunnelling diodes (RTDs)^12–14^, Schottky barrier diodes^15–17^ and heterostructure barrier varactor diodes^18^, and THz transistors such as heterojunction bipolar transistors^19,20^, high electron mobility transistors^21–23^, and silicon (Si) complementary metal–oxide–semiconductor devices^24–28^ have been reported. Various studies have been conducted to enhance the output power of the transmitter (Tx) and the sensitivity of the receiver (Rx) in the THz band. However, operation in the THz band is still challenging owing to the limitation of the device speed even though integrated-circuit technologies have been scaled down.

Coherent detection assisted by a local oscillator (LO) is an effective way to enhance the sensitivity of the Rx including an amplitude modulation (AM) system. For coherent detection, the frequency and phase of the LO should be aligned with those of the detected radio-frequency (RF) carrier signal or down-converted intermediate-frequency (IF) pilot signal^29^. One of the major methods for coherent detection at THz band is digital signal processing for the IF signal^30^. Another is synchronisation between the Tx and the Rx using a low-frequency reference signal with frequency multipliers for THz operations^31^. These systems are complicated and have a large power consumption.

For developing a simple synchronisation system between the Tx and the Rx, we can apply an injection-locking phenomenon to a self-oscillating mixer (SOM) as the Rx^32^, which is a self-oscillating electronic device with a non-linear current–voltage (*I*–*V*) characteristic that also acts as a mixer to demodulate a data signal. When the received carrier frequency from the Tx is set within the locking range of the LO frequency, the SOM will be injection-locked to the carrier signal, and coherent detection can be achieved in a single device. To develop an injection-locked SOM for the THz band, a THz fundamental oscillator is required.

Here, we propose to employ an RTD as the SOM^33,34^ for the THz coherent detector, which provides the fundamental oscillation in the THz band. The RTD has a negative differential conductance (NDC) region attributable to the quantum tunnelling effect in the *I*–*V* characteristic^35,36^. Oscillation occurs when the NDC compensates for the loss of the resonant circuit. An oscillator using an RTD was first realised in 1984 with an 18-GHz oscillation frequency at 200 K^37^; thereafter, the oscillation frequency was increased through an improved layer structure and a reduced parasitic capacitance. In 1991, 712-GHz oscillation was realised at room temperature^38^, and presently, a fundamental oscillation frequency of 1.98 THz, which is the highest oscillation frequency of any electronic single oscillator to date, has been achieved at room temperature^39^. In addition, RTDs are capable of acting as high-sensitivity direct detectors because they have a strong non-linearity in their *I*–*V* characteristics^40^. Thus, RTDs can work as both the Tx and Rx in a single device by adjusting the applied bias voltage. In recent years, various applications using THz RTDs have been reported, including wireless communications^41–44^, sensing^45^, and imaging^46^. Real-time error-free (bit-error rate (BER) < 10^−11^) transmission at 9 Gbit/s in the 300-GHz band with a system using RTDs has previously been reported^43^. In that system, a direct detection was employed in the Rx. One of the factors limiting the data rate of wireless communications using an RTD is the Rx sensitivity. Therefore, if we enhance the sensitivity by coherent detection using an RTD as an injection-locked SOM, the data rate will be significantly increased.

In this paper, we first describe the operating principles of the proposed THz coherent detection system. We then show that the detected power can be improved by introducing this system through an investigation using circuit simulations. Next, we discuss the fabrication of an RTD oscillator that oscillates in the 300-GHz band and demonstrate its operation by experiment. The results show coherent detection characteristics and sensitivity enhancement compared to conventional direct detection. Finally, we apply this system to THz wireless communications and demonstrate high-speed error-free data transmission.

### Operating principles

**Figure 1:**
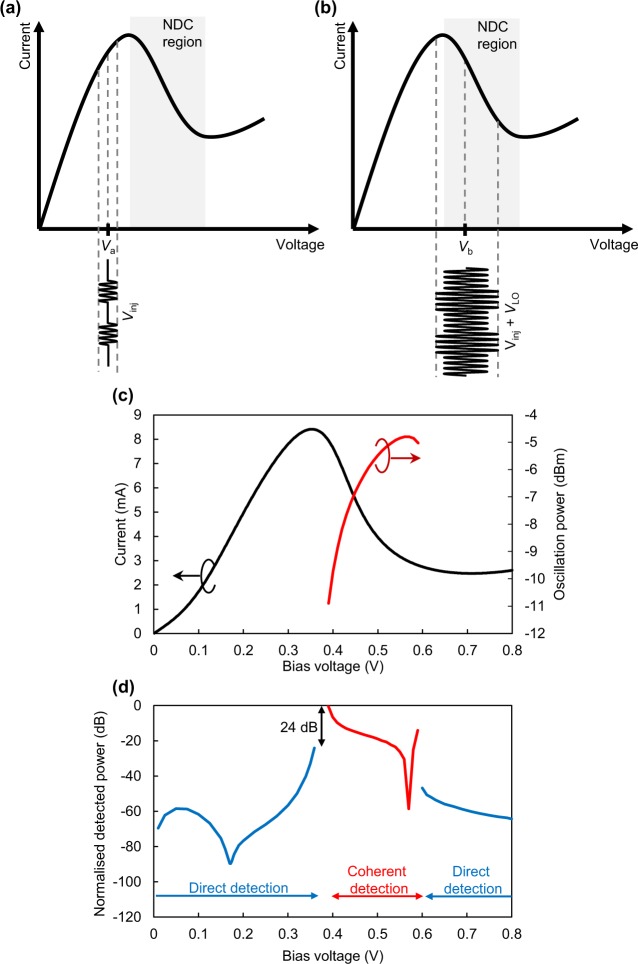
Relationship between the bias voltage and the received signals and the simulation results for the detected power. (**a**) Bias voltage in a conventional scheme set outside the NDC region. Direct detection is performed by the non-linearity. (**b**) Bias voltage in the proposed scheme set within the NDC region. RTD oscillation is locked to the received signals by using the injection-locking phenomenon. (**c**) *I*–*V* characteristics and oscillation power of the RTD oscillator. (**d**) Simulation results for the bias dependence of the detected power. With coherent detection, the detected power is improved by 24 dB compared to that with direct detection for an input power of −45 dBm and an oscillation power of −11 dBm.

In this section, we describe the operating principles of the coherent detection system. The configuration of the system is almost the same as that previously reported in ref. ^43^. An important difference is the bias voltage applied to the RTD for operation. Figure 1a,b show the relationships between the bias voltage and the received signals in a conventional direct system and the system proposed in this study. In a direct detection system, the sensitivity of the Rx is dependent upon the non-linearity of the *I*–*V* characteristics^47^. The non-linearity of the *I*–*V* characteristics of an RTD is the largest at the voltage for which the peak current is obtainable; thus, the largest sensitivity of the Rx might be obtained at that voltage. However, at this condition, the operation of the RTD becomes unstable, and the noise increases^48^. In addition, impedance matching at the baseband (BB) is more difficult close to the NDC area because the impedance of the RTD approaches infinity. Thus, when RTDs have been used as direct detectors, as shown in Fig. 1a, a somewhat smaller voltage to the voltage at the peak current is obtained.

In contrast, the SOM condition is fulfilled when the RTD is biased within the NDC region, as shown in Fig. 1b. The RTD oscillator acts as an LO signal and can contribute to the enhancement in the received signal. However, the oscillation from the LO will not be locked by the received signal. The sensitivity might be enhanced, but doing so will make the detected signals unstable. To solve this issue, we employ an injection-locking phenomenon. When the carrier frequency received from the Tx is set within the locking range of the RTD oscillator, these signals will be synchronised, and the received signals are added to the oscillation signal in-phase. These signals are mixed by the non-linearity in the NDC region, and the BB signals are obtained. Generally, the LO power and non-linearity affect the Rx efficiency in coherent detection. Both the oscillation power (i.e. LO signal) and the non-linearity of the RTD have a bias-voltage dependency; therefore, optimisation between them has to be achieved.

We estimated the bias-voltage dependence of the detected power using a circuit simulation (see Methods). In the simulation, we used the RTD model shown in ref. ^13^. Figure 1c shows the *I*–*V* characteristics and oscillation power of the RTD, and Fig. 1d shows the bias dependence of the detected power when the received signal power is assumed to be −45 dBm and the LO power is assumed to be −11 dBm. The variation in the detected power is attributed to the non-linearity of the *I*–*V* characteristics, which also affects the oscillation power in the case of coherent detection. In direct detection, as mentioned above, the detected power becomes the highest in the vicinity of a turning point. In coherent detection, the highest power is obtained in the vicinity of a turning point, at which the oscillation power is the lowest. The characteristics are the similar to reported with a self-oscillating tunnel-diode mixer^49^. On the boundary between direct and coherent detection, the oscillation becomes unstable and the detected signal cannot be measured. The largest detected power obtainable within the coherent detection regions is 24 dB higher than that obtainable in the direct detection regions. The simulation results show that an improvement in the Rx sensitivity is possible using coherent detection instead of direct detection.

### Fabrication and experimental demonstration

**Figure 2:**
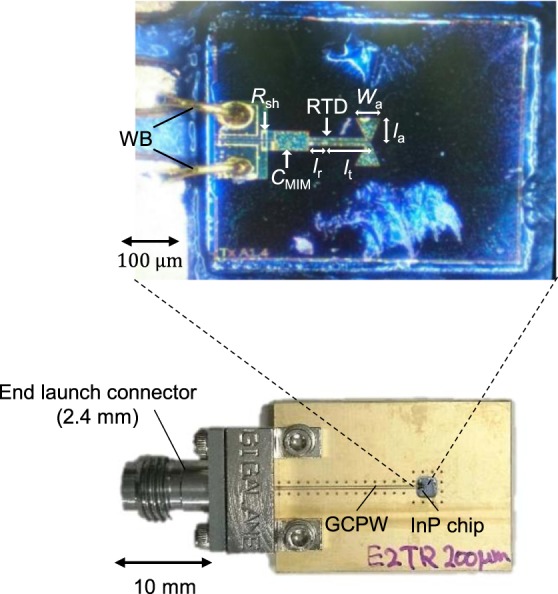
Fabricated RTD device. Photograph of the device composed of an RTD, a bow-tie antenna, a CPS, a MIM, a shunt resistor, and BB circuit with a GCPW and an end-launch connector.

Figure 2 shows a photograph of the fabricated RTD oscillator, which consists of an RTD, an antenna, a coplanar stripline (CPS), a metal–insulator–metal (MIM) capacitor (*C*_MIM_), and shunt resistor (*R*_s_). Its design is based upon the device in ref. ^43^. However, the size of the electrode pad is reduced to decrease its influence on the radiation pattern. The details of the design are described in Supplementary Section 2. In addition, the circuit for BB providing the bias voltage and data signal through the wire bonding (WB) has also been improved compared to previous publications^43^. The circuit used in this study is a grounded coplanar waveguide (GCPW) with a 2.4-mm end-launch connector attached. A Si super-hemispherical lens with a 6-mm radius and 2.16-mm offset length is attached to the back surface of the chip. The directivity of the bowtie antenna is enhanced by the Si lens^43^ to be around 27 dBi^50^, which was estimated from the measured radiation pattern^51^. We prepared 4 devices for the experiments. Their detail characteristics and the figures in which each device was used are described in Supplementary Section 3.

**Figure 3:**
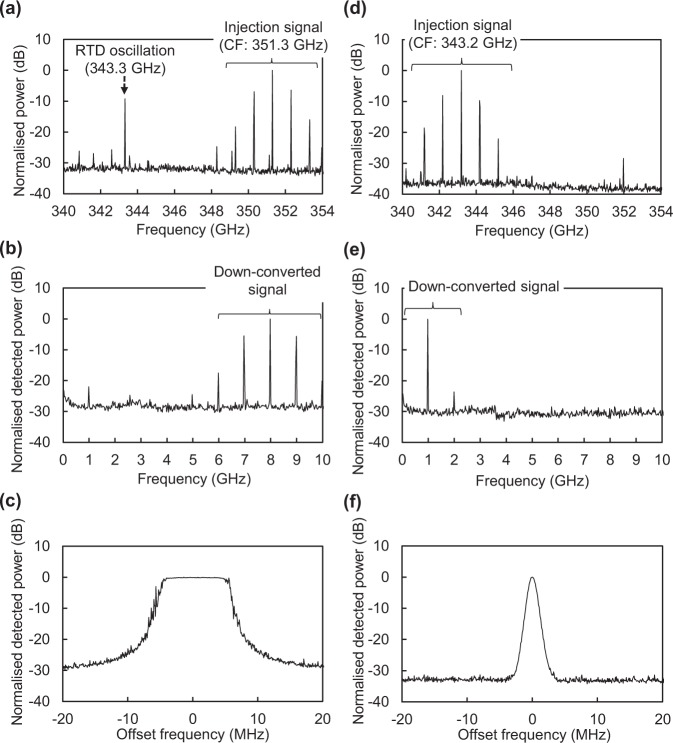
300-GHz band and detected signal spectra when receiving AM signals. (**a**) 300-GHz band spectra with a carrier frequency (CF) of 351.3 GHz. RTD oscillation is not locked to the carrier wave. (**b**) Detected signal spectra with a carrier frequency of 351.3 GHz. The spectra of the AM signals at the centre frequency of 8 GHz were observed. This is the result of self-oscillating mixing of the RTD oscillator. Since the carrier and RTD oscillation frequencies are different, we have heterodyne detection. (**c**) Results for the maximum hold values of the centre spectra of the detected signals with a carrier frequency of 351.3 GHz. (**d**) 300-GHz band spectra with a carrier wave frequency of 343.2 GHz. (**e**) Detected signal spectra with a carrier frequency of 343.2 GHz. (**f**) Results for the maximum hold values of the centre spectra of the detected signals with a carrier frequency of 343.2 GHz.

At first, we demonstrate coherent detection operation and an enhancement in the Rx sensitivity. We describe the details of the experimental setup in Methods. We use a multiplier on the Tx side and generate 300-GHz-band signals whose amplitudes are modulated with a 1-GHz sine wave. On the Rx side, we detect the AM signals with an RTD device. The bias voltage applied to the RTD was 600 mV, and the oscillation frequency was 343.3 GHz. In the experiment, we changed the carrier frequency to produce observed individual spectra when there was and was not injection locking. First, we set the transmitted carrier frequency to 351.3 GHz, a difference of 8 GHz from the RTD oscillation frequency. Figure 3a shows the spectra of the injected AM and RTD oscillation signals, and Fig. 3b shows the spectra of detected signals. In Fig. 3a, the spectra of the RTD oscillation signals and injected signals could be independently observed. Thus, the RTD oscillation signals are not locked to the injected signals. In Fig. 3b, the spectra of the AM signals of the centre frequency of 8 GHz can be observed. This is the result of self-oscillating mixing of the RTD oscillator. Since the carrier frequency and RTD oscillation frequency are different, we have heterodyne detection. Figure 3c shows the results of the maximum hold values of the centre spectra of the down-converted signals after 30 s by using the MAXHOLD mode of a spectrum analyser. Frequency fluctuations of 9.5 MHz are observed. This is probably because the carrier waves and RTD oscillation are not synchronised, and the frequency difference between them is not constant. Next, we set the carrier frequency to 343.2 GHz, a difference of 0.1 GHz from the RTD oscillation frequency. Figure 3d shows the spectra of the injected AM and RTD oscillation signals, and Fig. 3e shows the spectra of detected signals. In Fig. 3d, it is only possible to observe the spectra of the injected signals, and the spectra of the RTD oscillation signals at 343.3 GHz in Fig. 3a cannot be observed. This suggests that the RTD oscillation signals are injection-locked to the 343.2-GHz carrier wave. In Fig. 3e, 1-GHz signals, which are the modulating signals, are obtained. Since the carrier and RTD oscillation frequencies match, homodyne detection is performed. Figure 3f shows the results for the maximum hold values of the detected signal by the same method used before. The frequency fluctuations of the detected signals are suppressed to 0.6 MHz or below. This is probably because the carrier waves and RTD oscillation are synchronised by injection locking, and there is no frequency deviation between them. The noise level of Fig. 3f is reduced compared with that of Fig. 3c, which is consistent in the discussion on the injection locking in oscillators^52^, whereas the NDC can enhance the noise^53–55^. The theoretical model taking into account the quantum transport and injection locking in RTD oscillators will be required for further quantitative analysis of the noise. The locking range of the system is discussed in Supplementary Section 5.

**Figure 4:**
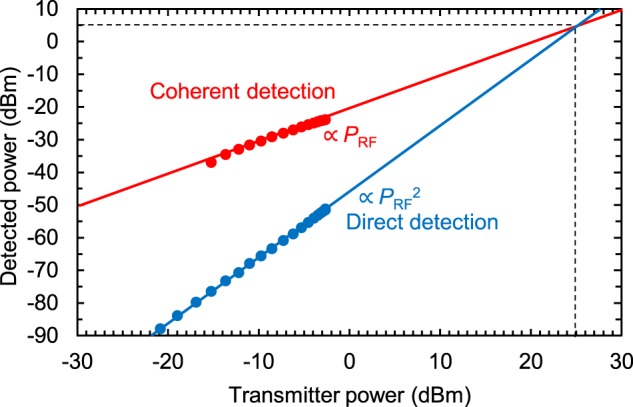
Relationship between the detected power and the Tx power. The detected power was improved by a maximum of 40 dB compared to conventional direct detection.

Next, we experimentally show that the detected power of the proposed coherent detection is enhanced in comparison with that of the direct detection. The experiment was performed by changing the bias voltage applied to the RTD; that is, the RTD was biased outside the NDC region to act as a direct detector or inside the NDC region to act as a coherent detector. We set the bias voltages to obtain the highest power in each region (Fig. 1d). Figure 4 shows the dependence of the detected power on the Tx power for the two RTD conditions. When the RTD is biased inside the NDC region, the detected power increases in proportion to the square of the Tx power, which is typical for square-law detection. On the other hand, when the RTD is biased outside NDC region, the detected power increases in proportion to the Tx power, which is a characteristic of coherent detection^29^. Under Tx power of −15 dBm, the coherent detection does not work since locking condition cannot be satisfied. The detected power of the coherent condition is larger compared to that of direct detection. The maximum difference between them is 40 dB at a Tx power of −15 dBm. These results show the effectiveness of the proposed scheme in terms of an enhancement in the Rx sensitivity. The conversion loss of this device is estimated to be around 10 dB (See Supplementary Section 6).

### Application to THz wireless communications

**Figure 5:**
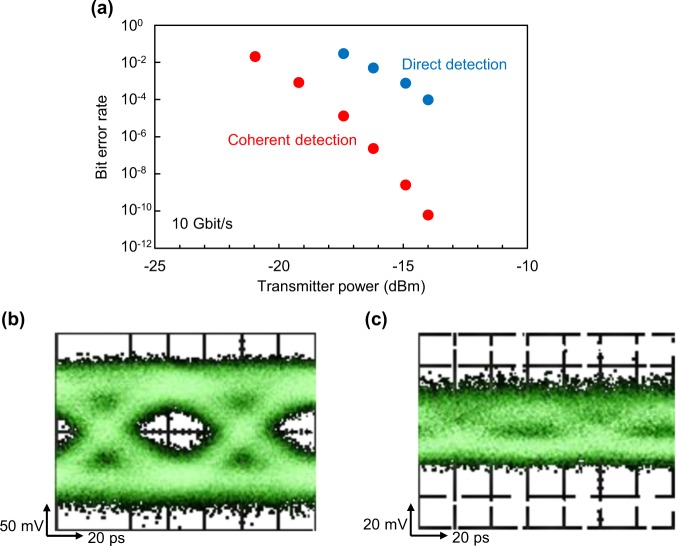
Results of the wireless communications experiment using a photo-mixing Tx. (**a**) Measured BER versus the Tx output power at 10 Gbit/s. Owing to the sensitivity enhancement, the BER is successfully reduced by coherent detection. (**b**,**c)** Eye diagram by direct and coherent detections at 27 Gbit/s for Tx power of -12 dBm, respectively.

We perform a THz communications experiment using the RTD coherent Rx in order to observe the impact of the Rx sensitivity. At first, we compared the transmission performance of coherent detection with that of direct detection, in which a free-running photonics-based Tx was employed. The details of the experimental setup are described in Methods. Figure 5a shows the dependence of the BER on the Tx output power at a data rate of 10 Gbit/s. Here, the carrier frequency was set to be equal to the RTD oscillation frequency for coherent detection. The RTD was biased inside the NDC region to act as a coherent detector, while a bias voltage outside the NDC region was applied for non-coherent direct detection. The BER decreases as the power increases owing to the increase in detected signal intensity. Thanks to the sensitivity enhancement by coherent detection, the BER is significantly reduced. Figure 5b,c show eye diagrams at a data rate of 27 Gbit/s. The amplitude difference of eye diagram between coherent and direct detections indicates that the sensitivity of the coherent detection is enhanced by about 15 dB compared with that of the direct one.

**Figure 6:**
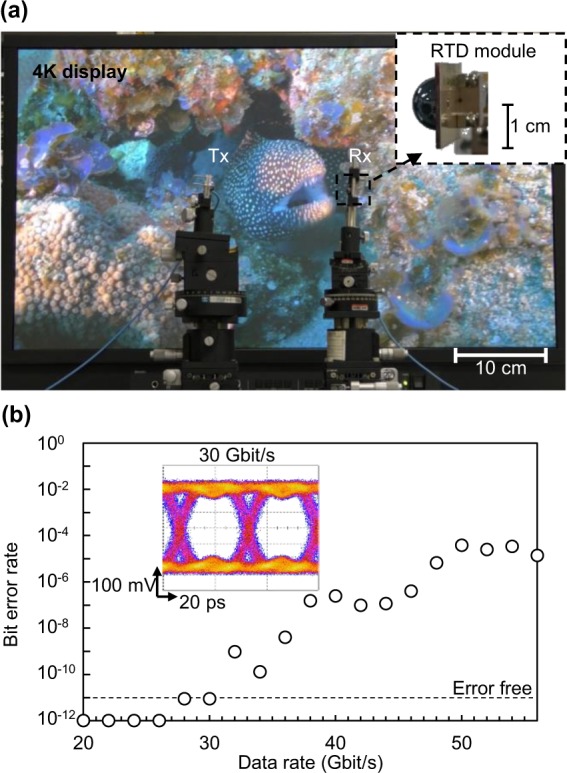
Results of the wireless communications experiment using an RTD Tx. (**a**) Photograph of 4 K video transmission using an RTD Tx and Rx. (**b**) Measured BER versus the data rate and the eye diagram at 30 Gbit/s. Error-free wireless transmission at 30 Gbit/s was achieved.

Finally, we apply the proposed coherent detection to an all RTD wireless communications system. In this system, amplitude-shift keying direct modulation is performed by changing the bias voltage applied to the RTD on the Tx side. We performed a wireless transmission experiment with uncompressed 4 K high-definition video^43^, as shown in Fig. 6a. The detailed setup and a movie are displayed in Supplementary Fig. S8a and Movie 1, respectively. The video was transmitted without interruption, indicating that an error-free condition was realised. Figure 6b shows the dependence of the BER on the data rate and the eye diagram measured at a data rate of 30 Gbit/s. Error-free transmission of a 30 Gbit/s signal is achieved with a clear eye diagram. This is the highest among real-time error-free wireless transmission based on all electronic devices without error correction to the best of our knowledge (see Supplementary Table S3). As the data rate increases, the BER increases, and the BER at a data rate of 56 Gbit/s is 1.39 × 10^−5^. The BB 3-dB bandwidth of the system is 19 GHz (Supplementary Fig. S8c). The data rate could be further increased by expanding the BB bandwidth.

## Conclusion

We proposed a simple coherent detection system in the THz band using an RTD oscillator. The proposed system was realised by an injection-locked RTD SOM. We demonstrated coherent detection characteristics and showed that the detected power was enhanced by 40 dB at maximum with the proposed system compared to conventional direct detection. We applied the system to wireless communications and showed that the BER was reduced compared to conventional direct detection. Moreover, we introduced the proposed coherent detection to an RTD-based THz wireless communications system and achieved error-free transmission at a data rate of 30 Gbit/s. Beyond wireless communications, the proposed simple and compact coherent THz detector can be applied to advanced imaging, sensing, and ranging systems, which would enable progress to be made in a wide range of fields in materials science, medicine, chemistry, biology, physics, astronomy, security, robotics, and motor vehicles.

## Methods

### Circuit simulation

For the calculation of the detected power, we used an RF circuit simulator, Keysight ADS. We used transient convolution to simulate the injection-locking phenomenon. The circuit model is shown in Supplementary Fig. S1. An antenna, a CPS, an MIM capacitor, and a shunt resistor were modelled by lumped components to simplify the calculation. The injection signals were modelled by a multi-tone source. We created AM signals (modulation frequency: 1 GHz, modulation ratio: 1) for the injection signals to calculate the detected power. In the calculation of the relationship between the detected power and the bias voltage shown in Fig. 1d, the carrier frequency was set to be equal to the RTD oscillation frequency at each bias voltage.

### Experimental setup: detection of am signals

A block diagram of the experimental setup is shown in Supplementary Fig. S5. A millimetre wave (36–40 GHz) from signal generator 1 was amplitude modulated by an electrical mixer with a 1-GHz sine wave from signal generator 2 and amplified using a 29-dB electrical amplifier. The signal was multiplied by nine times to generate THz signals at 324–360 GHz. A variable attenuator changed the Tx power. The THz signals were radiated into free space through a circular horn antenna. The transmission distance was about 20 cm. On the Rx side, the THz signals were detected by the RTD device. The detected signals were amplified by a 30-dB electrical amplifier and measured using spectrum analyser 1.

We measured the frequencies of the signals from the multiplier and RTD using a mixer system to observe the injection-locking phenomenon. The signals transmitted through the RTD module were received by a circular horn antenna, which was connected to the mixer. An RTD oscillation signal radiating from the backside of the RTD module was also received by the horn antenna. These signals were down-converted to an IF by the mixer for display on spectrum analyser 2, where the frequency and received power were measured.

### Experimental setup: wireless communications using a photonics-based tx

A schematic of the experimental setup is shown in Supplementary Fig. S7. For the Tx, infrared-light signals from two wavelength-tuneable lasers were combined by a coupler, and the intensity was modulated by an electro-optic modulator (EOM). The resultant signal was then amplified by an Er-doped optical fibre amplifier (EDFA). The EOM was driven by a pseudo-random binary sequence (PRBS) from a pulse pattern generator (PPG) with a repetition length of 2^15^ – 1. The modulated optical signals were down-converted to THz signals by using a uni-travelling-carrier photodiode (UTC-PD) module. The THz signals were radiated into free space through a circular horn antenna. The transmission distance was about 2 cm. On the Rx side, the THz signals were detected by the RTD device. The demodulated signals were amplified by a low-noise amplifier. The eye diagram and BER were measured using an oscilloscope and an error detector, respectively. Two commercial equalisers enhanced the bandwidths of the BB circuit.

### Experimental setup: wireless communications using rtd tx

A schematic of the experimental setup is shown in Supplementary Fig. S8b. For the Tx, a random data signal (2^15^ – 1 PRBS) from a PPG was applied to the RTD with a direct-current (DC) bias voltage through a bias tee. The Tx used in the experiment was of the same type as the device used in ref. ^43^. The transmitted power was 28 µW, and the transmission distance was about 7 cm. On the Rx side, the THz signals were detected by the RTD. The demodulated signal was amplified and measured with the same setup of the photonics-based Tx. A commercial equaliser was used for the enhancement of bandwidths of the BB circuit from 9 GHz to 19 GHz (Supplementary Fig. S8c).

## Supplementary information

Supplementary Information - Terahertz coherent receiver using a single resonant tunnelling diode

Supplementary Movie - Terahertz coherent receiver using a single resonant tunnelling diode
