# Supplementary material for: Terahertz coherent receiver using a single resonant tunnelling diode
Source: Sci Rep. 2019 Dec 2;9:18125. doi: 10.1038/s41598-019-54627-8 (PMC6889129; doi:10.1038/s41598-019-54627-8)
Supplement: Supplementary file 1 — Supplementary Information - Terahertz coherent receiver using a single resonant tunnelling diode [file 41598_2019_54627_MOESM1_ESM.pdf]

Supplementary Information

Terahertz coherent receiver using a single resonant tunnelling diode

YOUSUKE NISHIDA[1,2*], NAOKI NISHIGAMI[1*], SEBASTIAN DIEBOLD[1], JAEYOUNG KIM[2], MASAYUKI FUJITA[1*], and TADAO NAGATSUMA[1]

[1] *Graduate School of Engineering Science, Osaka University, 1-3 Machikaneyama, Toyonaka, Osaka 560-8531, Japan*

[2] *ROHM Co., Ltd., 21 Saiin Mizosaki, Ukyo, Kyoto 615-8585, Japan*

***e-mail: fujita@ee.es.osaka-u.ac.jp**

*These authors contributed equally to this work

1. Circuit Model

Figure S1 shows the circuit model of the resonant tunnelling diode (RTD) devices for the simulations[S1]. An antenna, a coplanar stripline (CPS), a metal–insulator–metal (MIM) capacitor (C_{MIM}), and a shunt resistor (R_{sh}) were modelled by lumped components to simplify the calculation. We used a circuit simulator, Keysight ADS. To simulate the injection-locking phenomenon, transient convolution was employed.

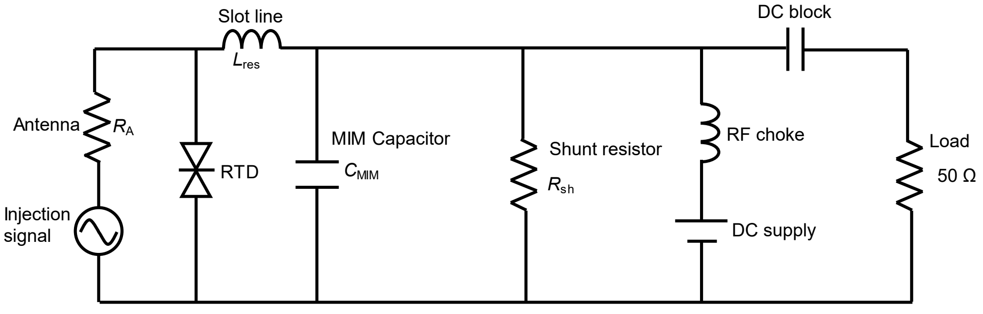

Figure S1 | Circuit model. We assumed that $R_A = 150\ \Omega$, $L_{res} = 17$ pH, $C_{MIM} = 120$ fF, and $R_{sh} = 35\ \Omega$, and the electrode area of the RTD = 1.4 μm^2.

2. Layout of the Fabricated RTD Circuit Chip

Figure S2 shows the design of the RTD device. A bowtie antenna is used as a radiator. A CPS provides impedance matching between the antenna and the RTD. A C_{MIM} provides a

short for a terahertz (THz) signal and an open circuit for a direct-current (DC) signal. A R_{sh} suppresses parasitic oscillation. We designed the oscillation frequency, which is determined by the capacitance of the RTD and the impedance of the resonator, using the circuit model[S1] described in Supplementary Section 1. We fabricated four RTD devices of which parameters are shown in Table S1, for making various oscillation frequencies. The oscillation frequency can be slightly tuned by changing the bias voltage of RTD, as mentioned in Supplementary Section 3.

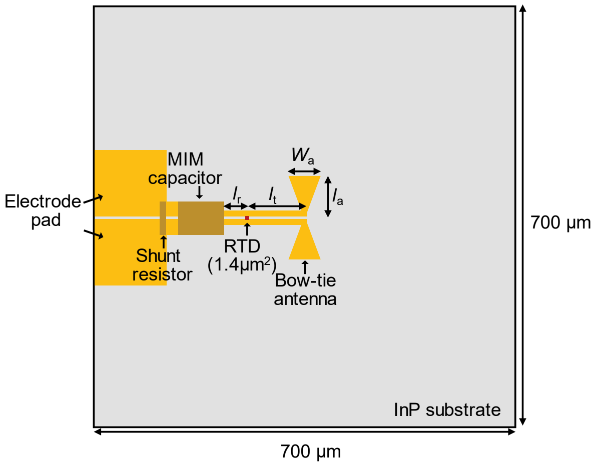

Figure S2 | Layout of the RTD chip.

Device No.	l_a (μm)	W_a (μm)	l_t (μm)	l_r (μm)
1	60	50	90	40
2	60	50	90	35
3	60	50	50	35
4	75	50	80	20

Table S1 | The parameters of the fabricated RTD circuits

3. Characteristics of the Fabricated RTDs

Figure S3 shows the RTD layer structure[S2]. The device No.4 has the thicker emitter spacer (20 nm) compared with other devices (No.1–3, 10 nm), which increases the operating voltage and current density, but the other layers are same among No.1–4. Mesa size of these devices are different due to process variation. Figure S3 shows the current–voltage (*I*–*V*) and oscillation frequency for each device and Table S2 summarized the operating voltage and oscillation frequency used in this study. It also includes estimated mesa size

and peak current density calculated from the Fig. S2. We employed the larger peak current devices (No. 3 and No. 4) for all RTD wireless communication experiments in order to have the larger oscillation power.

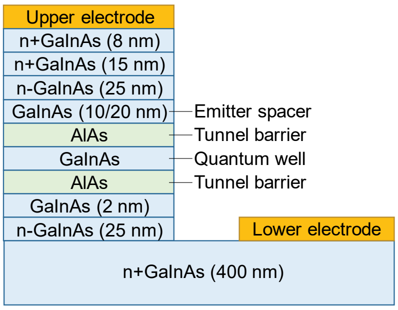

Figure S3 | Typical RTD layer structure.

Device No.	Type	Operation voltage (V)	Oscillation frequency (GHz)	Mesa size (μm^2)	Current density (mA/μm^2)	For
1	Rx	0.47-0.67	322.5-343.3	0.74	7.4	Fig. 3
2	Rx	0.45-0.66	322.2-350.3	0.74	8.2	Fig. 5
3	Rx	0.46-0.68	333.7-347.8	1.37	7.3	Fig. 4 & Fig. 6
4	Tx	0.64-0.90	336.5-360.2	1.10	13.5	Fig. 6

Table S2 | Fabricated devices and figures in which each device was used.

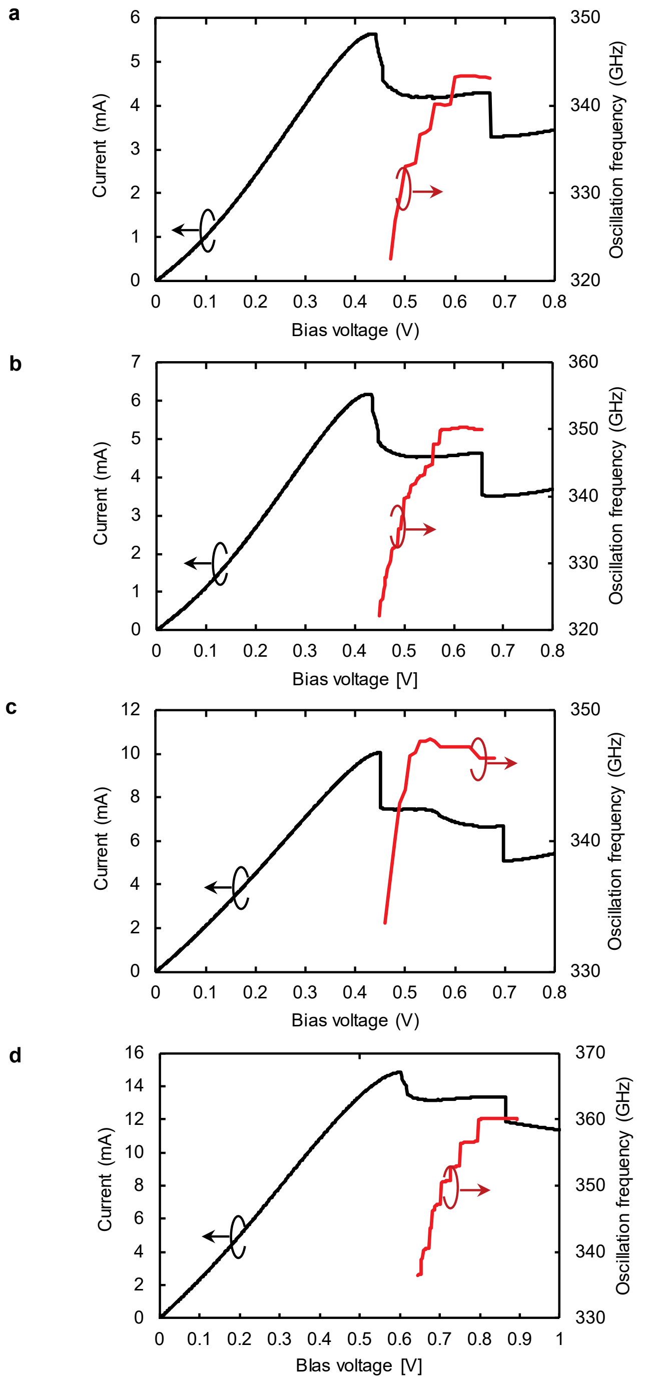

Figure S4 | The I-V and oscillation frequency of the fabricated device. (**a**) No.1, (**b**) No.2, (**c**) No.3, (**d**) No.4.

4. Experimental Setup: Detection of Amplitude-Modulated Signals

A block diagram of the experimental setup is shown in Figure S5. A millimetre wave (36–40 GHz) from signal generator 1 was amplitude-modulated (AM) by an electrical mixer with a 1-GHz sine wave from signal generator 2 and amplified using a 29-dB driver amplifier. The signal was multiplied by nine times to generate THz signals at 324–360 GHz. A variable attenuator changed the Tx power. A circular horn antenna was used to generate THz signals in free space that were detected by an RTD Rx. The detected signals were amplified by a 30-dB electrical amplifier and measured using spectrum analyser 1. We also measured the frequencies of the signals from the multiplier and RTD using a mixer system to observe the injection-locking phenomenon. The signals transmitted through the RTD were received by a circular horn antenna, which was connected to the mixer. An RTD oscillation signal radiating from the backside of the RTD module was also received by the horn antenna. These signals were down-converted to an intermediate frequency (IF) by the mixer using a LO for display on spectrum analyser 2, where the frequency and received power were measured.

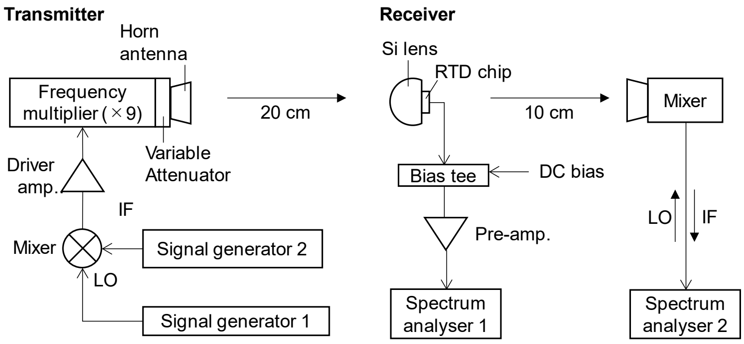

Figure S5 | Experimental setup for the detection of AM signals.

5. Frequency Locking Range Measurement

The operation frequency range to induce the injection locking phenomenon of the RTD oscillator was measured in an experiment. Figure S6a shows a block diagram of the experimental setup. The locking phenomenon was observed by the RTD oscillation spectrum using a mixer system. The oscillation frequency of the Rx was set to 352.6 GHz. The frequency of the multiplier Tx was changed from 338 GHz to 364 GHz. In theory, the

locking range can be expressed as

$$\Delta f = \frac{f_0}{Q} \cdot \sqrt{\frac{P_\mathrm{i}}{P_\mathrm{o}}} \tag{S1}$$

where f_0 is the oscillation frequency without locking, Q is the quality factor of the oscillator circuit, P_i is the injected power, and P_o is the oscillator output power[S3]. Equation S1 indicates that the locking range increases as the injected power increases. Fig. S6b shows the locking range with various Tx output powers. As the Tx power increases, the locking range is increased over 10 GHz. The experimental results show good agreement with the theoretical curve for Q = 6 and P_o = -15 dBm, taking into account the propagation loss (20 dB) of the Tx power to calculate P_i.

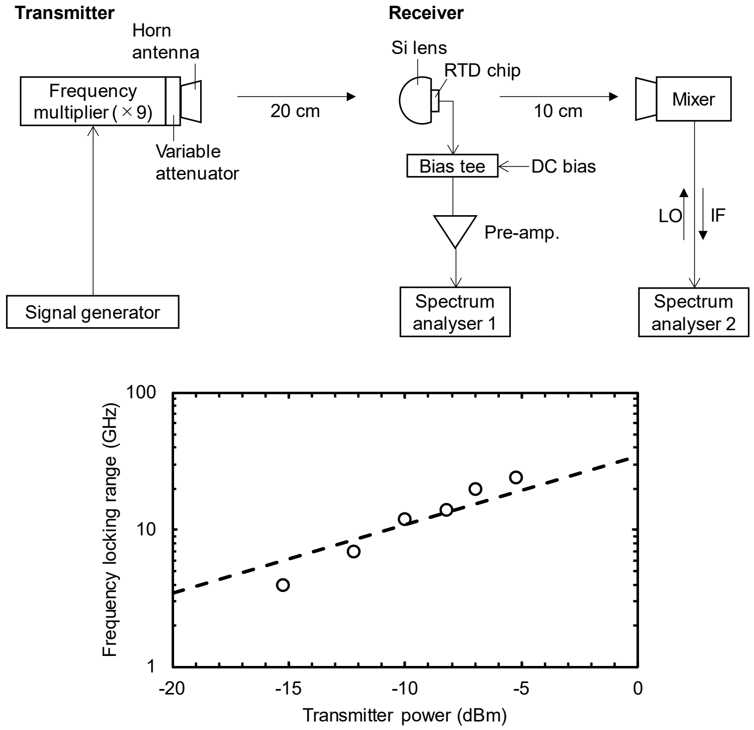

Figure S6 | Frequency locking range measurement. **(a)** Experimental setup. **(b)** Measurement plot as a function of the Tx power with the theoretical curve.

6. Conversion Loss Estimation

Conversion loss (L_C) is generally defined as[S4]

$$L_\mathrm{C} = 10\log\frac{P_\mathrm{RF}}{P_\mathrm{IF}}\ \mathrm{dB} \tag{S2}$$

where P_{RF} and P_{IF} are available RF input power and IF output power, respectively. L_{C} can be also calculated using the relationship between amplitude modulation(AM) signal and detected signal. When a mixer receives an AM signal and an LO signal which is coherent with the received RF carrier signal, both the upper sideband (USB) signal and the lower sideband (LSB) signal are down converted to the detected signal. Using equation (S2), L_{C} can be calculated by

$$L_{\mathrm{C}} = 10\log\frac{P_{\mathrm{USB}} + P_{\mathrm{LSB}}}{P_{\mathrm{Det}}}\ \mathrm{dB} \tag{S3}$$

where P_{USB} is the power of the USB signal, P_{LSB} is the power of the LSB signal and P_{Det} is the detected power. When the modulation index (m) is 1, the RF carrier signal power (P_0) is described as[S5]

$$P_0 = 2(P_{\mathrm{USB}} + P_{\mathrm{LSB}}). \tag{S4}$$

Using equation (S3) and (S4),

$$L_{\mathrm{C}} = 10\log\frac{P_0}{2P_{\mathrm{Det}}}\ \mathrm{dB}. \tag{S5}$$

Therefore, if we can know P_0 and P_{Det}, L_{C} can be calculated. P_0 can be estimated by the relationship between square-law detection and homodyne detection.

When a diode receives an AM signal, the diode voltage is expressed as

$$v_0(1 + m\cos\omega_{\mathrm{m}}t)\cos\omega_0 t \tag{S6}$$

where v_0 is the amplitude of the RF carrier signal, ω_{m} is the modulation frequency and ω_0 is the RF carrier frequency. In the case of square-law detection, the detected current is expressed as

$$\frac{m}{2}G_{\mathrm{d}}' v_0^2 \cos\omega_{\mathrm{m}}t \tag{S7}$$

where G_{d}' is the reciprocals of the second derivative of the diode's I-V curve[S4]. Next, we consider homodyne detection with injection-locked self-oscillating mixer. When the RF signal (amplitude: v_{r}, frequency: ω_{r}) and LO signal (amplitude: v_{l}, frequency: ω_{l}) are combined in a diode mixer, the current in the mixer can be expressed as[S4]

$$\frac{G_{\mathrm{d}}'}{2}(v_{\mathrm{r}}\cos\omega_{\mathrm{r}}t + v_{\mathrm{l}}\cos\omega_{\mathrm{l}}t)^2. \tag{S8}$$

When the amplitude of free-running oscillation is v_{OSC}, the amplitude of oscillation injection-locked with the RF carrier signal is described as

$$(v_{\mathrm{OSC}} - v_0)\cos\omega_0 t \tag{S9}$$

because the oscillation amplitude is limited by the negative conductance region. Using equation (S6), (S8) and (S9), the current in the mixer is expressed as

$$\frac{G_{\mathrm{d}}'}{2}\{v_0(1+\cos\omega_m t)\cos\omega_0 t + (v_{\mathrm{OSC}} - v_0)\cos\omega_0 t\}^2. \tag{S10}$$

The down-converted current is calculated as

$$\frac{m}{2}G_{\mathrm{d}}' v_0 v_{\mathrm{OSC}}\cos\omega_m t. \tag{S11}$$

When $v_0 = v_{\mathrm{OSC}}$, equation (S11) is described as

$$\frac{m}{2}G_{\mathrm{d}}' v_0^2\cos\omega_m t. \tag{S12}$$

Therefore, the detected power obtained by both detection schemes should be equal when the power of the RF carrier signal and free-running oscillator is the same. The RF carrier signal power can be estimated using the oscillation power.

In the experimental result, the detected power of each detection scheme would become the same at the Tx power of 25 dBm. At this Tx power, the RTD oscillation power and the RF carrier signal power received by the RTD should be equal, if the nonlinearities of each bias voltage are the same. Therefore, the RF carrier signal power received by the RTD can be estimated to be -11 dBm when the RTD oscillation power of -11 dBm is assumed, as described in the section of Operating Principles in main manuscript. At the Tx power of 25 dBm, the detected power would be -24 dBm, after removing the effect of the 29 dB amplifier. Using equation (S5),

$$L_{\mathrm{C}} = -11 - (-24 + 3) = 10 \ \ \mathrm{dB}. \tag{S13}$$

The conversion loss of the RTD device acting as a coherent detector is roughly estimated to be 10 dB.

7. Experimental Setup: Wireless Communications Using a Photonics-Based Tx

A schematic of the experimental setup is shown in Supplementary Figure S7. For the Tx, infrared-light signals from two wavelength-tuneable lasers were combined by a coupler, and the intensity was modulated by an electro-optic modulator (EOM). The resultant signal was then amplified by an Er-doped optical fibre amplifier (EDFA). The EOM was driven by a pseudo-random binary sequence (PRBS) from a pulse pattern generator (PPG) with a repetition length of $2^{15} - 1$. The modulated optical signals were down-converted to

a THz signal by using a uni-travelling-carrier photodiode (UTC-PD) module. The THz signals were radiated into free space through a circular horn antenna. The transmission distance was about 2 cm. On the Rx side, the THz signals were detected by the RTD device. The demodulated signals were amplified by a low-noise amplifier. The eye diagram and bit-error rate (BER) were measured using an oscilloscope and error detector, respectively. Two commercial equalisers enhanced the bandwidths of the baseband (BB) circuit.

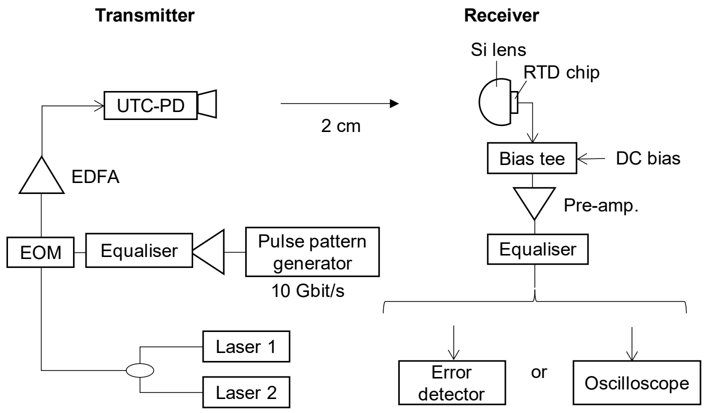

Figure S7 | Experimental setup for wireless communications using a photonics-based Tx.

8. Experimental Setup: Wireless Communications Using an RTD Tx

Figure S8a shows a block diagram of the 4K video transmission system. For the Tx, a 4K recorder (HyperDeck Studio Pro by Blackmagic Design) was used, which provided an uncompressed 4K video signals with a data rate of ~6 Gbit/s. The data signals were applied to the RTD with a DC bias voltage through a bias tee. The transmitted power was 28 μW, and the transmission distance was about 15 cm. On the Rx side, the THz signals were detected by the RTD. The demodulated signals were amplified by an amplifier and reshaped by a limiting amplifier. A converter (Blackmagic Design) converted a 6G-SDI signal into an HDMI signal. The signals were then input to a television.

Fig. S8b shows a block diagram of the BER and eye-diagram measurement setup. For the Tx, instead of using the 4K recorder, a PPG, which provides a random data signal (2^{15} – 1 PRBS), was used. The transmission distance was about 7 cm. On the Rx side, the THz signals were detected by the RTD. The demodulated signals were amplified and measured with the same setup as the photonics-based Tx. An equaliser enhanced the bandwidths of the BB circuit from 9 GHz to 19 GHz, as shown in Fig. S8c.

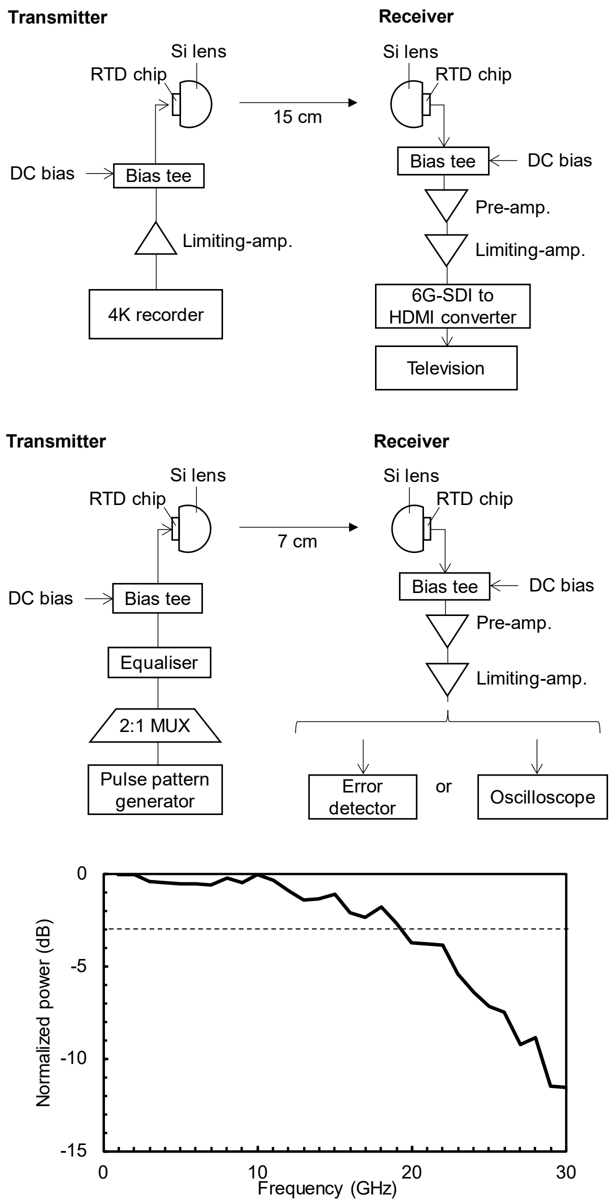

Figure S8 | Wireless communications system. **(a)** Experimental setup for 4K data transmission. **(b)** Wireless communications experiment setup. **(c)** Frequency response of the system baseband.

9. Comparison of the Wireless Communications Performance Using Various Devices

Table S3 summarises the achieved bit rates (≥10 Gbit/s) and measured BERs for various wireless communications systems and carrier frequencies using only electronic devices, including high-electron-mobility transistors (HEMTs), complementary metal–oxide–semiconductor (CMOS) transistors, Schottky barrier diodes (SBDs), and RTD devices as

the Tx and Rx. The BER reported here was directly measured in real time by a BER tester. Various modulation formats were employed, such as amplitude shift keying (ASK), phase shift keying (PSK), and quadrature amplitude modulation (QAM). The practical error-free condition is a BER less than 10^{-11}, which is sufficient for practical video transmission without interruption. For error-free transmission, the bit rate of the system proposed in this study is the highest among all systems based on all electronic devices without an error correction process to the best of our knowledge

Ref. No.	S6	S7	S8	S9	S10	S11	S12	S13	S14	S15	S16	S17	S18	S19	S20	S21	S22	This Study
Tx	HEMT	HEMT	CMOS	CMOS	HEMT	CMOS	CMOS	HEMT	RTD	RTD	CMOS	CMOS	CMOS	RTD	CMOS	CMOS	HEMT	RTD
Rx	HEMT	HEMT	CMOS	CMOS	HEMT	CMOS	CMOS	HEMT	SBD	RTD	CMOS	CMOS	CMOS	SBD	CMOS	CMOS	HEMT	RTD
Bit rate (Gbit/s)	11	10	10	10	96	10	56	20	22	12	12.5	42.2	32	56	27.8	120	100	30
Carrier Frequency (GHz)	123	220	135	130	240	130	68, 102	300	490	286	130	60	294	490	60	70, 105	287	350
Bit error rate	$<10^{-11}$	1.6×10^{-9}	N/A	$<10^{-12}$	N/A	1×10^{-11}	N/A	$<10^{-11}$	$<10^{-12}$	2.3×10^{-3}	$<10^{-10}$	N/A	N/A	$<10^{-3}$	7×10^{-4}	N/A	N/A	$<10^{-11}$
Modulation Format	ASK	ASK	ASK	ASK	8PSK	ASK	16QAM	ASK	ASK	ASK	ASK	64QAM	16QAM	ASK	16QAM	16QAM	16QAM	ASK

Table S3 | Comparison of previously reported wireless communications systems and that proposed in this study.

References

S1 Diebold, S. *et al.* Modeling and simulation of terahertz resonant tunneling diode-based circuits. *IEEE Trans. Terahertz Sci. & Technol.* **6**, 716–723 (2016).

S2 Shiode, T., Mukai, T., Kawamura, M. & Nagatsuma, T. Giga-bit wireless communication at 300 GHz using resonant tunneling diode detector. in *Asia-Pacific Microw. Conf.*, (2011).

S3 Adler, R. A study of locking phenomena in oscillators. *Proc. IRE* **34**, 351–357 (1946).

S4 Pozar, M. D. in *Microwave Engineering* 2nd Edn, 549–568 (Wiley, 1998).

S5 Gagliardi, R. M. in *Introduction to Communications Engineering*, 46–52 (Wiley, 1978).

S6 Kosugi, T. *et al.* 120-GHz Tx/Rx chipset for 10-Gbit/s wireless applications using 0.1-μm-gate InP HEMTs. in *IEEE Compd. Semicond. Integr. Circuit Symp.* (2004).

S7 Kallfass, I. *et al.* All active MMIC-based wireless communication at 220 GHz. *IEEE Trans. Terahertz Sci. Technol.* **1**, 477–487 (2011).

S8 Zhang, B., Xiong, Y. Z., Wang, L. & Hu, S. A switch-based ASK modulator for 10 Gbps 135 GHz communication by 0.13 μm MOSFET. *IEEE Microw. Wirel. Components Lett.* **22**, 415–417 (2012).

S9 Byeon, C. W., Yoon, C. H. & Park, C. S. A 67-mW 10.7-Gb/s 60-GHz OOK CMOS transceiver for short-range wireless communications. *IEEE Trans. Microw. Theory Tech.* **61**, 3391–3401 (2013).

S10 Kallfass, I. *et al.* 64 Gbit/s transmission over 850 m fixed wireless link at 240 GHz carrier frequency. *J. Infrared, Millimeter, Terahertz Waves* **36**, 221–233 (2015).

S11 Fujishima, M. *et al.* Tehrahertz CMOS design for low-power and high-speed wireless communication. *IEICE Trans. Electron.* **E98.C**, 1091–1104, (2015).

S12 Tokgoz, K. K. *et al.* A 56 Gb/s W-band CMOS wireless transceiver. in *IEEE Int. Solid-State Circuits Conf. (ISSCC) Dig. Tech.* (2016).

S13 Song, H. *et al.* Demonstration of 20-Gbps wireless data transmission at 300 GHz for KIOSK instant data downloading applications with InP MMICs. in *IEEE MTT-S Int. Microw. Symp.* (2016).

S14 Oshima, N., Hashimoto, K., Suzuki, S. & Asada, M. Wireless data transmission of 34 Gbit/s at a 500-GHz range using resonant-tunnelling-diode terahertz oscillator. *Electron. Lett.* **52**, 1897–1898 (2016).

S15 Diebold, S. *et al.* High-speed error-free wireless data transmission using a terahertz resonant tunnelling diode transmitter and receiver. *Electron. Lett.* **52,** 1999–2001 (2016).

S16 Dolatsha, N. *et al.* A compact 130 GHz fully packaged point-to-point wireless system with 3D-printed 26dBi lens antenna achieving 12.5Gb/s at 1.55pJ/b/m. in *IEEE Int. Solid-State Circuits Conf. (ISSCC) Dig. Tech.* (2017).

S17 Pang, J. *et al.* A 128-QAM 60 GHz CMOS transceiver for IEEE802. 11ay with calibration of LO feedthrough and I / Q imbalance. in *IEEE Int. Solid-State Circuits Conf. (ISSCC) Dig. Tech.* 424–426 (2017).

S18 Hara, S. *et al.* A 32 Gbit/s 16QAM CMOS receiver in 300 GHz band. in *IEEE MTT-S Int. Microw. Symp.* (2017).

S19 Oshima, N., Hashimoto, K., Suzuki, S. & Asada, M. Terahertz wireless data transmission with frequency and polarization division multiplexing using resonant-tunneling-diode oscillators. *IEEE Trans. Terahertz Sci. Technol.* **7**, 2813–2814 (2017).

S20 Daneshgar, S. *et al.* A 27.8 Gb/s 11.5 pJ/b 60 GHz transceiver in 28nm CMOS with polarization MIMO. in *IEEE Int. Solid-State Circuits Conf. (ISSCC) Dig. Tech.* (2018).
S21 Tokgoz, K. K. *et al.* A 120 Gb/s 16QAM CMOS millimeter-wave wireless transceiver. in *IEEE Int. Solid-State Circuits Conf. (ISSCC) Dig. Tech.* (2018).
S22 Hamada, H. *et al.* 300-GHz, 100-Gb/s InP-HEMT wireless transceiver using a 300-GHz fundamental mixer. in *IEEE MTT-S Int. Microw. Symp.* (2018).
